# Effects of combination therapy indacaterol/glycopyrronium versus tiotropium on moderate to severe COPD: evaluation of impulse oscillometry and exacerbation rate

**DOI:** 10.1186/s40248-017-0105-4

**Published:** 2017-09-27

**Authors:** Antonio Molino, Francesca Simioli, Anna Agnese Stanziola, Mauro Mormile, Maria Martino, Maria D’Amato

**Affiliations:** Respiratory Department Federico II University- A.O. “Dei Colli”, Via D Fontana, 134, 80128 Naples, Italy

**Keywords:** COPD, Impulse oscillometry lung function, Pulmonary function test, Small airway obstruction

## Abstract

**Background:**

Small airways are considered the major site of airflow limitation in COPD. Impulse oscillometry (IOS) is a forced oscillation technique, which provides passive measurement of lung mechanics. It can differentiate small airway from large airway obstruction and is more sensitive than spirometry for peripheral airway disease. In this study the efficacy of the combination of Indacaterol/Glycopirronium (IND/GLY) versus Tiotropium on airway resistance (R5, R20, R5–20), lung reactance (X) and resonant frequency in moderate to severe COPD patients has been evaluated. We also evaluated inspiratory capacity (IC), forced expiratory volume in 1 s (FEV_1_), forced vital capacity (FVC), exacerbation rate and quality of life.

**Methods:**

Forty patients were monitored with forced oscillation technique and spirometry. Patients were randomized in 2 groups: 20 received fixed dose once daily Indacaterol/Glycopyrronium (Group A) and 20 received single Tiotropium (Group B).

The oscillometry parameters were the measure of resistance in the airways at 5 Hz (R5), at 20 Hz (R20) and the lung reactance (X).

**Results:**

There was a statistically significant difference between pre-dosing at V_1_ and at follow up visits in R_5_, R_20_ and X values in patients receiving dual bronchodilation but not in control group. Pre-dosing IC value at follow up visits in patients receiving dual bronchodilation had a statistical significant variation.

**Conclusions:**

The “new” bronchodilator combination LABA/LAMA significantly reduces bronchial obstruction in small airways too. The oscillometry demonstrated greater sensitivity compared with spirometry for monitoring outcome measures of airway obstruction and the effect of long-term therapy.

## Background

Chronic Obstructive Pulmonary Disease (COPD) is a common, preventable and treatable disease characterized by persistent respiratory symptoms and airflow limitation due to airway and/or alveolar abnormalities [1]. At present, the diagnosis and staging of COPD is difficult, as spirometry alone is unable to evaluate the severity of this disease because early pathological changes in COPD are localized within small airways with diameter < 2 mm to 4 mm. Static lung hyperinflation is often one of the significant challenges in patients with COPD. It is characterised by a decrease in the elastic recoil of the lungs with a premature closure of small airways leading to air trapping [2]. The impact on lung function parameters is expressed by an increase in functional residual capacity (FRC) and a progressive decrease in inspiratory reserve volume and inspiratory capacity (IC) [3]. Spirometry involves a forced expiratory manoeuvre, which may not be the ideal test to detect subtle improvements in airway calibre in COPD due to effort-dependent small airways closure. Furthermore, FEV_1_ mainly measures the degree of obstruction in large and intermediate airways [4, 5] while COPD is a disease mainly residing in the peripheral airways. Other lung function assessments are necessary to describe the disease pattern. Impulse oscillometry (IOS) is easier to perform in COPD patients [6]. It is an effort - independent test performed during normal quiet breathing, thereby obviating expiratory small airways closure and measuring the frequency - dependent airway resistance and reactance (X) [7].

Tiotropium is a long-acting muscarinic antagonist, with a 24-h persistent bronchodilator effect thus given once daily [8]. Actually, according to the Global Initiative for Chronic Obstructive Lung Disease (GOLD) guidelines, the bronchodilation therapy (anticholinergic and β_2_-agonists) is central to the pharmacological interventions for COPD. We can use single or double (combination of β_2_ agonist and antimuscarinic drug) bronchodilator agent as maintenance therapy for patients with mild to very severe COPD, depending on symptoms severity and exacerbation frequency.

Combining bronchodilators with different mechanism and duration of action may increase the degree of bronchodilation with a lower risk of side-effects compared to increasing dosage of a single bronchodilator [1, 9]. Fixed dose combinations (FDCs) provide potent bronchodilation versus single agents, with some advantage in terms of convenience and simplicity compared with combinations administered via separate inhalers.

There is evidence from prospective clinical studies indicating greater improvement in lung function with LABA/LAMA combination therapy compared with increasing the dose of a single bronchodilator in patients with moderate-to-severe COPD [9]. The once-daily fixed-dose combination of indacaterol (IND, a LABA) with glycopyrronium (GLY, a LAMA) 110/50 μg combines these two bronchodilators in a single inhaler and is approved for maintenance treatment of patients with moderate to very severe COPD.

However, there are limited data on the effects of the combination IND/GLY on small airways in patients with COPD.

In this study we evaluated the efficacy of the combination of IND/GLY versus Tiotropium alone on IOS parameters in patients with moderate to severe COPD. We also evaluated lung function including inspiratory capacity (IC), as well as exacerbation rate and quality of life.

## Methods

### Inclusion criteria

Male or female aged ≥40 years who had received a diagnosis of moderate-to- severe COPD according to Global Initiative for Chronic Obstructive Lung Disease guidelines [10], and had a post-bronchodilator forced expiratory volume in 1 s (FEV_1_) of ≥30% to <80% of predicted normal, [10] and a post-bronchodilator FEV_1_ to forced vital capacity (FVC) ratio of <0.7 smoking history >10 pack/years; and >1 ﻿exacerbation in the last 12 months.

### Exclusion criteria

Atopy; exacerbation within 4 weeks before screening, upper respiratory tract infection within 4 weeks before screening; other respiratory conditions such as haemoptysis, asthma, Idiopathic pulmonary fibrosis (IPF), lung cancer, recent history of rib fracture and pneumothorax.

### Subjects

Patient demographics and other baseline characteristics are shown in Table 1.

**Table 1:** Characteristics of patients

		Mean (SD) IND/GLY	Mean (SD) Tiotropium	Mean Total
Patients (M)		20 (17)	20 (18)	
Age		70.4 (7.83)	72.01 (6.79)	71.09
BMI		27.85 (5.54)	27.40 (6.02)	27.64
Years from COPD diagnosis		7.45 (5.5)	8.3 (4.6)	7.81
Smokers/Former smokers		3/17	1/14	4/31
GOLD Stage	B	8	7	15
	C	4	3	7
	D	8	5	13
FEV_1_	L	1.29 (0.33)	1.27 (0.39)	1.28
	%	52.26 (9.04)	52.13 (11.33)	52.20
FVC	L	2.21 (0.52)	2.18 (0.54)	2.20
	%	70.2 (9.4)	68.2 (9.5)	69.92
IT	%	52.6 (9.4)	56.2 (9.5)	54.5
IC	L	1.63 (0.59)	1.64 (0.61)	1.636
	%	68.61 (19.87)	69.16 (20.16)	68.85
Borg Dyspnea Scale		6.1 (2.33)	5.77 (2.12)	5.96
SGRQ		71.5 (15.97)	68.65 (16.32)	70.07

All patients attended the clinical laboratory for 4 study visits (every three months), from January 2015 to July 2016.

All subjects gave their written informed consent and the study was approved by Ethics Committee (SUN-AO Dei Colli, Naples, Italy).

### Study design

This was a 52-week randomized, open-label, parallel group study carried out in accordance with the Declaration of Helsinki.

### Protocol steps

Screening: assessment of COPD diagnosis and assessment of inclusion/exclusion criteria, prescription of a wash out period (Ultra-LABA 72 h, LABA and LAMA 48 h, SABA 8 h).

Visit 1 (T_0_): the patients filled in a questionnaire, performed lung function assessment, and oscillometry. Then they were randomized in a 1:1 ratio to receive either once daily IND/GLY 110/50 μg delivered via the Breezhaler® device (Novartis Pharma AG, Basel, Switzerland) or once- daily Tiotropium 2,5 μg Respimat® device (Boehringer Ingelheim, Ingelheim, Germany). The modality of drug taking was showed to patients and they assumed it. After 1 h and 3 h from that time lung function and oscillometry were performed again.

Visit 2 (3 months +/− 1 week), 3 (6 months +/− 1 week), 4 (12 months +/− 1 week): the patients filled in the questionnaire, repeated lung function assessment and oscillometry before the intake of daily dose of drug. They assumed the drug and performed again lung function assessment and oscillometry after 1 h and 3 h from the drug intake.

At each of the 4 study visits the patients undertook baseline tests (IOS, and spirometry), before inhalation therapy, after 1 h and 3 h from inhalation. Additional assessments were performed including the exacerbation analysis, and quality of life score by SGRQ.

Participants attended the department on the same time during each study visit.

#### Impulse oscillometry

The IOS system (IOS, Jaeger Master Screen, Jaeger Co, Wurzburg, Germany) noninvasively assesses respiratory mechanics without patient cooperation using small pressure oscillations generated at the mouth during spontaneous breathing. During the test, subjects firmly supported their cheeks while sitting with their neck in a comfortable neutral posture, wearing a nose clip, and tightly sealed their lips around the mouthpiece in order to stabilize the position of their tongue and to avoid buccal air leaks. Whole-breath, inspiratory (insp), and expiratory (exp) IOS measures of resistance measured the total airway resistance at an oscillation frequency of 5 Hz (R5), central airway resistance at 20 Hz (R20), peripheral resistance frequency dependence of resistance from 5 to 20 Hz (R5-R20), reactance at 5 Hz (X5), and area under the reactance curve (AX) and the resonant frequency (RF). Reported results are the average of 3 technically acceptable periods of 40 to 60 s of measure. Impulse oscillometry was performed in triplicate according to the manufacturer’s instructions [11–13].

#### Spirometry

Lung function measurements were performed according to manufacturer’s instructions and European Respiratory Society (ERS)/American Thoracic Society (ATS) recommendations [14, 15]. The reference values used were established by Crapo et al. [16].

The FEV_1_, FVC and the Inspiratory Capacity (IC), were measured using a dry wedge spirometer (Jaeger Co, Wurzburg, Germany). Baseline values at each visit were measured after at least 15 min of quiet rest, and the results (absolute values and percent predicted) were evaluated. Readings were again performed in triplicate, with the highest FEV_1_ recorded.

#### Exacerbations

A COPD exacerbation is defined as an acute worsening of respiratory symptoms which needs additional therapy, regardless the factors that cause it [17].

#### Quality of life

St. George’s Respiratory Questionnaire (SGRQ) is a disease-specific questionnaire designed to measure HRQoL in patients with chronic lung diseases. It consists of 17 questions divided into three categories: symptoms (wheeze, cough, and dyspnea), activities that are limited by the disease, and impact on the respondent’s social life and mental state. The scores ranges from 0 to 100, and lower values indicate better health status [18].

### Data analysis

The study was powered at 90% to detect a 0.1 kPa L-1 s difference in the primary outcome of trough R5, assuming a within subject standard deviation of 0.13 kPa L-1 s, and an alpha error of 0.05 (two-tailed) [19]. Students t-tests were used to compare treatment effects at baseline and after chronic dosing. The level of significance set at 0.05. Exacerbations’ analysis on Relative Risk assessment was based.

## Results

### Patients

Thirty-five patients completed the study (20 in Ind/Gly group and 15 in Tio group). Mean age was 71.03 years.

### Oscillometry

In Table 2 we compared pre dual bronchodilation values at V_1_ and V_4_.

**Table 2:** Pre dual bronchodilation values at V_1_ and V_4_

	V_1_ Pre dual bronchodilation	V_4_ Pre dual bronchodilation
R 5 Hz	0.74 ± 0.23	0.58 ± 0.13
R 20 Hz	0.45 ± 0.10	0.38 ± 0.06
X 5 Hz	-0.35 ± 0.16	-0.20 ± 0.09
IC	1.63 ± 0.59	2.04 ± 0.57
SGRQ	71.5 ± 15.97	58.25 ± 15.98

**Fig. 1:**
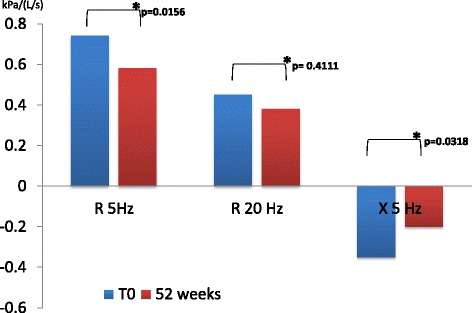
Resistance and reactance before and after dual bronchodilation

**Fig. 2:**
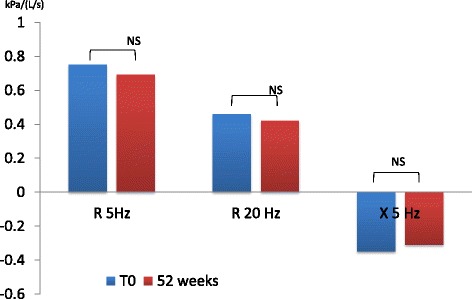
Resistance and reactance before and after tiotropium

We found a significant difference between pre-dosing R5Hz value at V_1_ and at follow up visits in patients receiving dual bronchodilation (mean difference − 0.16, 95% CI -0.2829 to −0.0371, *p* = 0.0122) but not in control group (mean difference − 0.07, 95% CI -0.2332 to 0.0984, *p* = 0.4111). R20Hz had a similar change (mean difference − 0.066, 95% CI -0.1255 to −0.0061, *p* = 0.0318). Reactance (X) also showed a difference at follow up only in patients receiving dual bronchodilation (mean difference 0.146, 95% CI 0.0586 to 0.2340, *p* = 0.0017) (Figs. 1 and 2)

.

### Spirometry

**Fig. 3:**
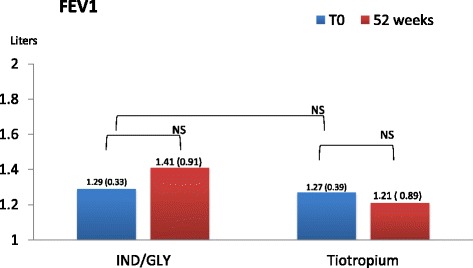
Pre-dosing Fev_1_(liters and DS) in two groups at T_0_ and after 52 weeks

**Fig. 4:**
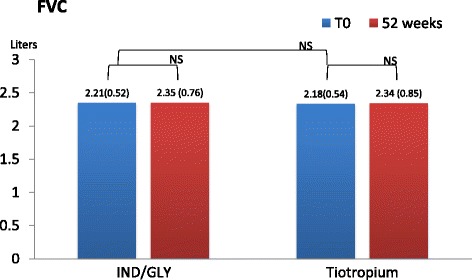
Pre-dosing FVC (liters and DS) in two groups at T_0_ and after 52 weeks

**Fig. 5:**
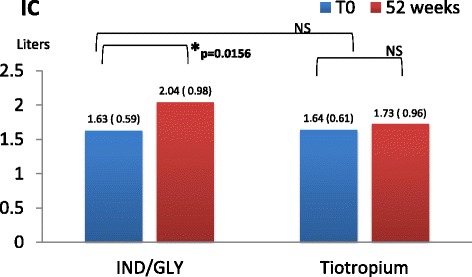
Pre-dosing IC in two groups at T_0_ and after 52 weeks

Lung function was investigated pre and after 1 h and 3 h from the assumption of inhaled therapy. At each visit we found that pre-dosing IC value in patients receiving dual bronchodilation had a statistical significant variation (mean difference + 0.41L, 95% CI 0.0505 to 0.7695, p = <0.05). Other parameters did not change neither after dual nor after mono-bronchodilation, FEV_1_ did not change significantly in cases (+0.12 L, *p* = 0.38), and in controls (−0.6 L, *p* = 0.76). FVC did not change significantly in cases (+0.0025 L, *p* = 0.99), and in controls (+0.01 L, *p* = 0.92) (Figs. 3, 4, and 5)

.

### Exacerbation rate

In patients receiving dual bronchodilation, we found a reduction of 66% of exacerbations (RR = 0,3383 (95% CI 0.1722 to 0.6649, *p* = 0.0017). Hospitalization rate was not different between the 2 groups, but among patients receiving mono-therapy there were more frequent exacerbators.

### Health status

**Fig. 6:**
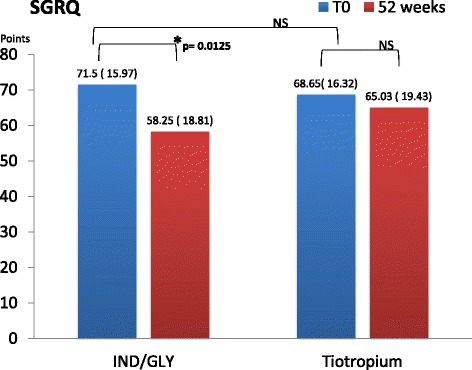
SGRQ mean difference and DS in two groups at T_0_ and after 52 weeks

Comparing V_1_and V_4_ SGRQ, we found a mean difference of −13.25 points (95% CI 3.024 to 23.4758, *p* = 0.0125) after dual bronchodilation therapy (Fig. 6)

.

## Discussion

In this study, the efficacy of a maintenance combination therapy with indacaterol/glycopyrronium versus tiotropium on IOS parameters in patients with moderate to severe COPD was studied. We demonstrated the efficacy of the combination of IND/GLY versus tiotropium on airway resistance (R5, R20, R5–20), lung reactance (X) and resonant frequency. All of them are small airways parameters. We don’t find a FEV_1_ and FVC statistical significance for the small sample size but in many other studies the once-daily fixed-dose combination IND/GLY 110/50 μg has been shown to significantly improve lung function and patient-reported outcomes (including dyspnea and health status) versus placebo, and versus Tiotropium [20, 21].

In the SPARK study, Wedzicha et al. [22] demonstrated that the dual bronchodilator IND/GLY was superior in preventing mild to severe COPD exacerbations compared with the single long-acting antimuscarinic bronchodilator glycopyrronium, with concomitant improvements in lung function and health status. Subsequently, the SHINE study [23] confirmed that dual bronchodilation with QVA149 provides additional therapeutic benefits compared to the mono-components indacaterol and glycopyrronium, as well as compared to tiotropium, the current gold standard of care, and placebo in patients with moderate-to-severe COPD. The authors demonstrated that improvement in the primary end-point, trough FEV_1_ was both statistically and clinically significant (considered to be 100 mL in COPD) over placebo and statistically significant versus active comparators approaching also clinical significance. IND/GLY also significantly improved TDI total scores compared with tiotropium [21–23].

It is generally accepted that FEV_1_ is not an optimal parameter for describing the complexity of COPD and that other lung function assessments are necessary to describe the disease pattern [24, 25].

Crisafulli et al. in an observational study on stable COPD patients demonstrated that there is a progressive increase in peripheral airway dysfunction among patients with different GOLD stages evaluated by both GOLD staging systems [25]. Furthermore, we demonstrated a strong relationship between small-airway dysfunction (SAD) as assessed by means of IOS and impact of disease [5].

Su-Gang Gong et al. observed that the IOS technology-related parameters have high sensitivity in detecting the slightly increased airway resistance that reflects a small airway airflow limitation [26, 27].

Kolsum U et al. evaluated 94 COPD and 58 out of them were followed up after 1 year . The authors confirmed that IOS measurements are related to the degree of airflow obstruction as measured by forced expiratory volume in 1 s (FEV_1_), and to the degree of hyperinflation. In addition, he found R5, X5 were all significantly associated (*p* < 0.05) with FEV_1_, sGaw, TLC, RV and IC but there was no statistically significant change in the FEV_1_, R5, X5 after 1 year. For the authors the changes in R5 and R20 did not significantly correlate with the changes in FEV_1_ [28].

It is known that bronchodilators increase airway diameters and decrease airway resistance making the pattern of airway obstruction more homogeneous in COPD patients [1]. The airway’s parameters of spirometry and the IOS are considered in the assessment of physiological changes in the large and small airways separately. Among these spirometric parameters, FEV_1_ is not well suited to assess the abnormalities in the small airways and is characterized as a large-airway parameter.

Raw is not frequently reported in studies evaluating the effect of bronchodilators in COPD. However, this parameter is suggested to be sensitive and to reflect airflow obstruction, particularly of the peripheral airways, more accurately than the FEV_1_/FVC ratio [29]. In assessing the acute functional effect of bronchodilators, specific Raw change-based criteria may be preferable to FEV_1_- or FVC-based criteria, being more closely related to bronchodilator-induced improvements in lung mechanics and dyspnea at rest.

Other parameters such as R10 and AX have also been shown to change in response to the bronchodilators [30, 31].

Manoharan et al., in randomized patients with moderate to severe COPD already taking ICS/LABA to receive add-on therapy in cross-over fashion with either TIO 18 μg od or ACL 322 μg subsequently bid for 2–3 weeks each, observed no significant differences between randomized treatments in any IOS or spirometry outcomes measured at trough after chronic dosing with TIO and ACL when used as triple therapy in patients with COPD [20].

In our study also the IC, the best parameter related to the reduction of hyperinflation [32], in patients receiving dual bronchodilation was improved. Improved IC is associated with improved exercise endurance and dyspnea [33, 34] and potentially improved long-term outcomes. Furthermore, the improvement in measures of hyperinflation is supported by the BRIGHT study [35] and recently by Salomon et al. [36].

## Conclusion

The oscillometry demonstrated greater sensitivity compared with spirometry for monitoring outcome measures of airway obstruction and the effect of long term therapy. This technique should facilitate the early optimization of therapy and a more personalized therapeutic approach for COPD patients. These data support the use of dual bronchodilator therapy to not only improve airways calibre (FEV_1_) but also decrease hyperinflation and its associated negative consequences in patients with COPD.

On the small airways, the combination IND/GLY induces a decrease of dynamic compression, a consequent decrease of dynamic hyperinflation leading to less dyspnea during exercise.

## Abbreviations

BMI: Body mass index
COPD: Chronic Obstructive Pulmonary Disease
FEV_1_: Forced expiratory volume in 1 s
FRC: Functional residual capacity
FVC: Forced vital capacity capacity
GOLD: Global Initiative for chronic obstructive Lung Disease
IC: Inspiratory capacity
IND/GLY: Indacaterol/Glycopirronium
IOS: Impulse oscillometry
LABA: Long-acting beta-agonist
LAMA: Long-acting muscarinic antagonist
SAD: Small-airway dysfunction
SGRQ: St. George’s Respiratory Questionnaire
X: Lung reactance
